# Supplementary material for: Causal Associations of Glaucoma and Age-Related Macular Degeneration with Cataract: A Bidirectional Two-Sample Mendelian Randomisation Study
Source: Genes (Basel). 2024 Mar 26;15(4):413. doi: 10.3390/genes15040413 (PMC11049509; doi:10.3390/genes15040413)
Supplement: Supplementary file 1 [file genes-15-00413-s001.zip › Supplementary File S1.pdf]

To reduce of concern according to population stratification, and enhance more robustly acknowledge, we performed additional analysis for association of cataract and glaucoma as Table S2. As for the data set, Finngen and UKB composed of European and meta-analysis results were appropriately used.

Table S2. Summary statistics of data source

Traits	Data source	No. of participants	Population	No. of Variants	Reference
Cataract_Fin	Finngen	372,386 (59,522 cases + 312,864 controls)	European	20,170,143	https://finngen.gitbook.io /documentation/v/r5/
Cataract_UKB	UKB	456,348 (14,867 cases + 441,481 controls)	European	11,831,932	[1]
Glaucoma_UKB	UKB	456,348 (654 cases + 455,694 controls)	European	11,831,932	[1]
Glaucoma_Meta	Meta-analysis for European	192,702 (15,229 cases + 177,473 controls)	European	12,713,176	[2]

Cataract_Fin, Cataract phenotype derived from Finngen; Cataract_UKB, Cataract phenotype derived from UK biobank; Glaucoma_UKB, Glaucoma phenotype derived from UK biobank; Glaucoma_Meta, Glaucoma phenotype derived from meta-analysis from European origin UK biobank lesser than 5%

In addition, heterogeneity and horizontal pleiotropy of instrumental variables were described as Table S3. When interpreting these results, it was found that the MR results (exposure: cataract _UKB, and glaucoma_Meta) should be interpreted focusing on the MR-Egger (SIMEX) method. In addition, when exposure is cataract_Fin and glaucoma_UKB, MR IVW method is ideal.

Table S3. Heterogeneity and horizontal pleiotropy of instrumental variables

Exposure	Outcome				Heterogeneity		Horizontal pleiotropy				
								MR-Egger		MR-Egger (SIMEX)	
		N	F	I2 (%)	p*	$p^{\dagger}$	$p^{\ddagger}$	Intercept, β (SE)	p	Intercept, β (SE)	p
Cataract_Fin	Glaucoma_UKB	38	53.67	80.68	0.251	0.219	0.261	0.009 (0.033)	0.778	0.008 (0.037)	0.823
Cataract_UKB	Glaucoma_Meta	11	50.32	69.11	<0.001	<0.001	<0.001	0.037 (0.044)	0.416	0.04 (0.05)	0.429
Glaucoma_UKB	Cataract_Fin	1	43.49	-	-	-	-	-	-	-	-
		2	35.97	85.28	0.174	-	-	-	-	-	-
Glaucoma_Meta	Cataract_UKB	45	64.89	87.14	0.002	0.004	0.002	-0.012 (0.007)	0.105	-0.014 (0.008)	0.098

N, number of instruments; F, mean F statistic; IVW, inverse-variance weight; MR, mendelian randomization; PRESSO, pleiotropy residual sum and outlier; SIMEX, simulation extrapolation; β, beta coefficient; SE, standard error

***Cochran's Q test from inverse-variance weight**

† Rucker's Q' test from MR-Egger

‡MR-pleiotropy residual sum and outlier global test

Cataract_Fin, Cataract phenotype derived from Finngen; Cataract_UKB, Cataract phenotype derived from UK biobank; Glaucoma_UKB, Glaucoma phenotype derived from UK biobank; Glaucoma_Meta, Glaucoma phenotype derived from meta-analysis from European origin UK biobank lesser than 5%

The results of MR analysis performed in the similar method as in the main text were obtained (Figure S1). No causal relationship of cataract on glaucoma was discovered (*Ps*> 0.05, Figure S1 and Table S4). However, significant causal associations of glaucoma on cataract were observed [*P*=0.012, IVW for exposure: glaucoma_UKB and outcome: Cataract_Fin; *P*=0.038 in MR-Egger (SIMEX) for exposure: glaucoma_Meta and outcome: Cataract_UKB]. The genetic connection between cataract on glaucoma for each SNP is represented by scatter plots with no correlations, whereas glaucoma effects on cataract were significant positive correlation (Figure S2).

Figure S1. Forest Plot for Association of Cataract and Glaucoma

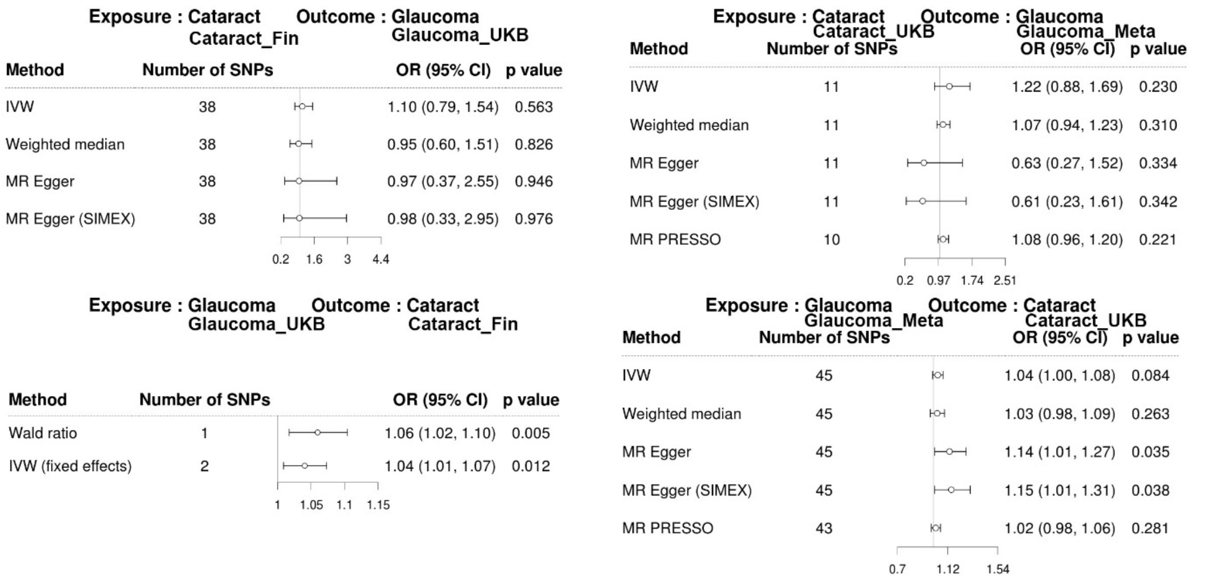

Cataract_Fin, Cataract phenotype derived from Finngen; Cataract_UKB, Cataract phenotype derived from UK biobank; Glaucoma_UKB, Glaucoma phenotype derived from UK biobank; Glaucoma_Meta, Glaucoma phenotype derived from meta-analysis from European origin UK biobank lesser than 5%

Table S4. Estemates from MR methods for the association between cataract and glaucoma

Exposure	Outcome	Methods	Parameter	N	Odds ratio (95% CI)	p-value
Cataract_Fin	Glaucoma_UKB					
		IVW	Estimate	38	1.10 (0.79, 1.54)	0.563
		Weighted median	Estimate		0.95 (0.60, 1.51)	0.826
		MR-Egger	Intercept		1.01 (0.95, 1.08)	0.778
			Slope		0.97 (0.37, 2.55)	0.946
		MR-Egger (SIMEX)	Intercept		1.01 (0.94, 1.08)	0.823
			Slope		0.98 (0.33, 2.95)	0.976
		MR-PRESSO	Estimate			
Cataract_UKB	Glaucoma_Meta					
		IVW	Estimate	28	1.22 (0.76, 1.98)	0.407
		Weighted median	Estimate		1.07 (0.92, 1.25)	0.378
		MR-Egger	Intercept		1.04 (0.95, 1.13)	0.416
			Slope		0.72 (0.18, 2.79)	0.635
		MR-Egger (SIMEX)	Intercept		1.04 (0.94, 1.15)	0.429
			Slope		0.68 (0.15, 3.17)	0.626
		MR-PRESSO	Estimate	25	1.05 (0.93, 1.19)	0.439
Glaucoma_UKB	Cataract_Fin					
		Wald ratio	Estimate	1	1.06 (1.02, 1.10)	0.005
		IVW	Estimate	2	1.04 (1.01, 1.07)	0.012
Glaucoma_Meta	Cataract_UKB					
	ukb	IVW	Estimate	45	1.04 (1.00, 1.08)	0.084

Weighted median	Estimate		1.03 (0.98, 1.09)	0.263
MR-Egger	Intercept		0.99 (0.97, 1.00)	0.105
	Slope		1.14 (1.01, 1.27)	0.035
MR-Egger (SIMEX)	Intercept		0.99 (0.97, 1.00)	0.098
	Slope		1.15 (1.01, 1.31)	0.038
MR-PRESSO	Estimate	43	1.02 (0.98, 1.06)	0.281

Cataract_Fin, Cataract phenotype derived from Finngen; Cataract_UKB, Cataract phenotype derived from UK biobank; Glaucoma_UKB, Glaucoma phenotype derived from UK biobank; Glaucoma_Meta, Glaucoma phenotype derived from meta-analysis from European origin UK biobank lesser than 5%

Figure S2. Scatter plots of MR tests assessing the bidirectional effect of cataract and glaucoma

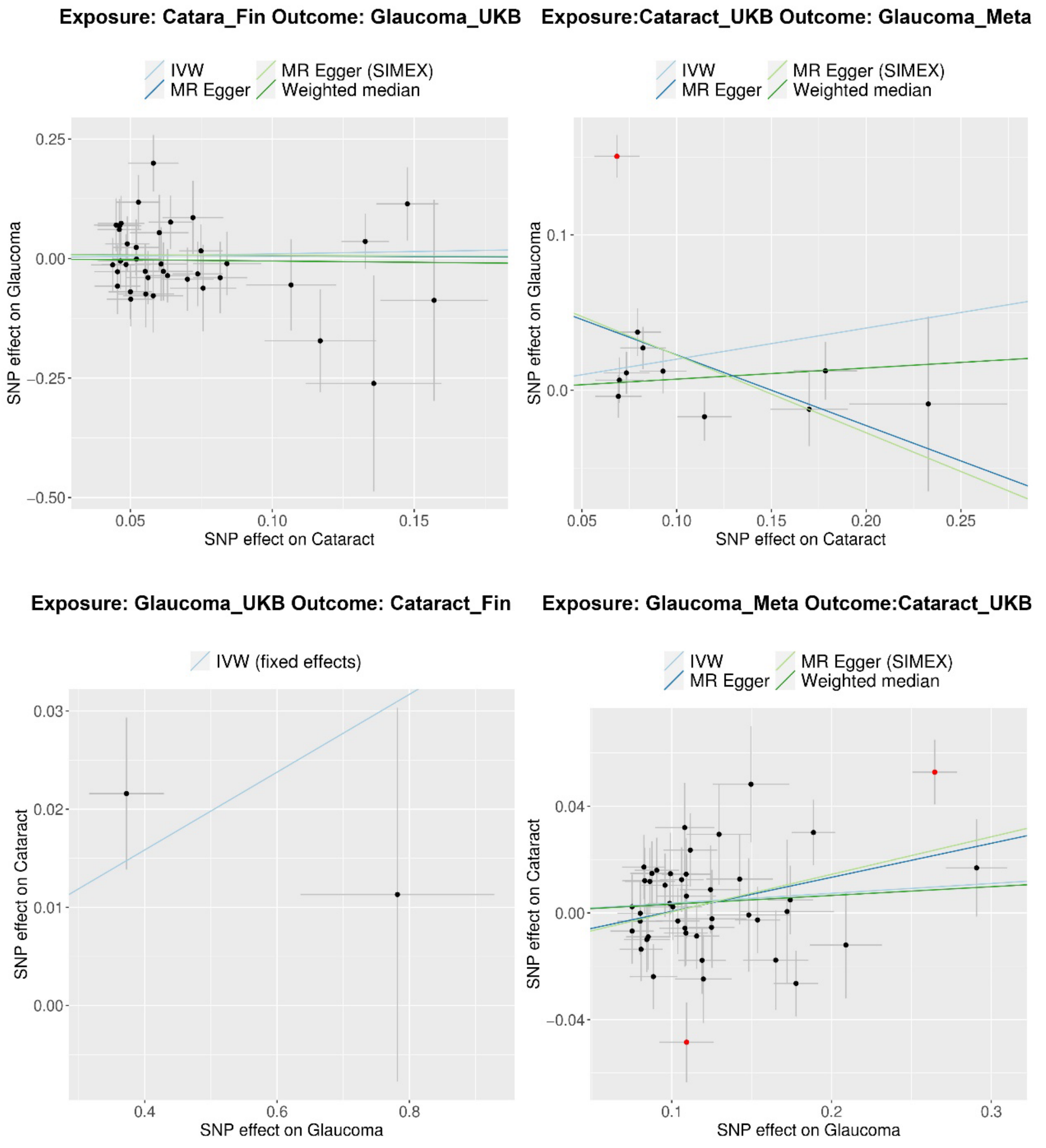

Light blue, light green, dark blue, and dark green regression lines represent the IVW, MR–Egger (SIMEX), MR–Egger, and weighted median estimate, respectively. Red dots indicate outliers based on the MR–PRESSO. When outliers were present, MR-PRESSO results were generated, and other MR methods were analysed without outlier

removal. MR, Mendelian randomization; SNP, single nucleotide polymorphism; IVW, inverse-variance weight; SIMEX, simulation extrapolation

References

1. Jiang, L.; Zheng, Z.; Fang, H.; Yang, J. A generalized linear mixed model association tool for biobank-scale data. *Nat Genet* **2021**, *53*, 1616-1621, doi:10.1038/s41588-021-00954-4.
2. Gharahkhani, P.; Jorgenson, E.; Hysi, P.; Khawaja, A.P.; Pendergrass, S.; Han, X.; Ong, J.S.; Hewitt, A.W.; Segre, A.V.; Rouhana, J.M.; et al. Genome-wide meta-analysis identifies 127 open-angle glaucoma loci with consistent effect across ancestries. *Nat Commun* **2021**, *12*, 1258, doi:10.1038/s41467-020-20851-4.
